# 2203. ‘I always feel like, somebody's watching me’--Multimodal Antimicrobial Stewardship Supports Antibiotic Prescribing for Common Infections within the UNC Health Virtual Practice

**DOI:** 10.1093/ofid/ofad500.1825

**Published:** 2023-11-27

**Authors:** Ashley H Marx, Nikolaos Mavrogiorgos, Amir Barzin

**Affiliations:** University of North Carolina Medical Center, Chapel Hill, North Carolina; UNC Medical Center; UNC School of Medicine, Chapel Hill, North Carolina; UNC Health Virtual Practice; UNC School of Medicine, Chapel Hill, North Carolina

## Abstract

**Background:**

Telehealth presents a new setting for antimicrobial stewardship interventions. Retrospective, disease-state-specific antibiotic review may be an effective mode of analyzing data and providing feedback to prescribers. The purpose of this project was to determine if prescriber-specific and composite team feedback were effective in maintaining adherence to preferred antibiotic selection and duration.

**Methods:**

In July 2022, multimodal educational materials were distributed to UNC Health Virtual Practice providers to promote the use of preferred antibiotics and the shortest effective durations for common infections.

Encounter notes during the study period (6/1/2022 - 4/30/2023) were searched to determine eligible encounters corresponding urinary tract infections (UTI), skin and soft tissue infections (SSTI), pneumonia, and sinusitis. Eligible encounters with linked prescriptions were analyzed to determine whether antibiotic selection(s) and duration of therapy matched clinic-established best practices. The proportion of prescriptions corresponding to best practice for each infection type was calculated for each visit type, and for each provider.

Feedback consisted of provider-specific memos from the medical director on antibiotic selection and duration; composite team performance was shared with team members at provider meetings and senior leadership in administrative reports.

**Results:**

The frequency of included visits was: sinusitis (393, 88%), SSTI (25, 6%), UTI (16, 4%), and pneumonia (11, 3%). The proportion of antibiotics corresponding to a firstline agent for each visit type were high for each included infection (range 72.6-100%; Table 1). Adherence to recommended duration of therapy was variable (range 12.5-100%). Six of 14 total prescribers had visits audited in both quarters. Among those, 5 of 6 increased or maintained personal performance from quarters 1 and 2 (Table 2).

**Figure:**
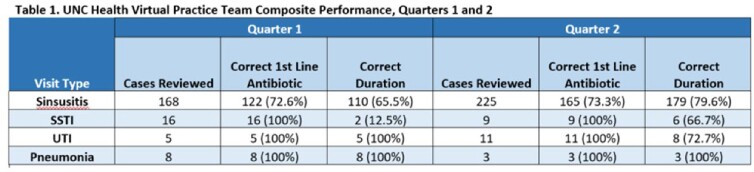

**Figure:**
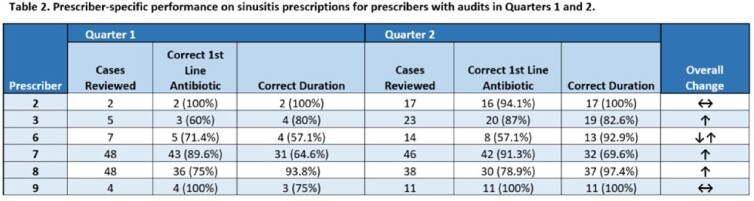

**Conclusion:**

Education followed by quarterly prescriber-specific and team feedback was associated with high rates of adherence to prescription of preferred agents. Use of the shortest effective duration of therapy presents additional opportunity for improvement. Targeted interventions for sinusitis provide the greatest stewardship opportunity for the UNC Health Virtual Practice. .

**Disclosures:**

**All Authors**: No reported disclosures

